# Supplementary material for: Prevalence and determinants of fertility care-seeking among women experiencing delayed conception in North India: a cross-sectional study
Source: Front Reprod Health. 2026 Mar 19;8:1772807. doi: 10.3389/frph.2026.1772807 (PMC13044071; doi:10.3389/frph.2026.1772807)
Supplement: Supplementary file 1 [file Table1.docx]

**Prevalence and determinants of fertility care-seeking among women with delayed conception in Northern India: a cross-sectional study**

**Short title:** Determinants of care-seeking among women with delayed conception in North India

Barsha Gadapani Pathak,^1,3^ Gitau Mburu,^2^ Ndema Habib,^2^ Rita Kabra,^2^ James Kiarie,^2^ Ranadip Chowdhury,^1^ Neeta Dhabhai,^1^ Sarmila Mazumder^1^*

1 Society for Applied Studies, New Delhi, India

2 UNDP-UNFPA-UNICEF-WHO-World Bank Special Program of Research, Development and Research Training in Human Reproduction (HRP) Department of Sexual and Reproductive Health and Research, World Health Organization, Geneva, Switzerland.

3 Centre for Intervention Science in Maternal and Child Health, Centre for International Health, Department of Global Public Health and Primary Care, University of Bergen, Bergen, Norway*,*

*Corresponding author

Email: [sarmila.mazumder@sas.org.in](mailto:sarmila.mazumder@sas.org.in)

**CONTENT:**

**Supplementary Table 1:** Proportion of women seeking care for delayed pregnancy, source of care and treatment/management received.

**Supplementary Figure 1:** Scree plot of eigen values after factor loading

**Supplementary Table 2:** Multivariable model representing the determinants of care-seeking for delayed conception from all sources of care versus formal and mixed sources of care among study participants

**Supplementary Table 1: Proportion of women seeking care for delayed pregnancy, source of care and treatment/management received.**

| Proportion of women who sought care from the different sources to assist to conceive (n=1530) | **N(%)** |
| --- | --- |
|  | **1069 (69.9)** |
| **Sources of care among who sought care** | **n = 1069** |
| **Traditional Healer** | |
| Number of women who consulted traditional doctors consulted | 384 (35.82)) |
| Mean (SD) traditional doctor consulted | 1.49 (1.13) |
| Number of times each traditional doctor consulted | |
| Median (IQR) | 3 (2 to 7) |
| Range | 1 to 70 |
| Treatment received |  |
| Advice | 241 (62.76) |
| Undergoing fertility diagnostic testing (diagnosis) | - |
| Ovulation induction | - |
| Insemination | - |
| Surgery | 1 |
| Assisted Reproduction Technology (ART) | - |
| Drugs | 346 (90.10) |
| Counselling | 58 (15.10) |
| None of these | 1 (0.26) |
| Others (folk, traditional and herbal remedies) | 7 (1.82) |
| **General doctor** | |
| Number of general doctors consulted | 174 (16.23) |
| Mean (SD) general doctor consulted | 1.24 (0.62) |
| Number of times each general doctor consulted | |
| Median (IQR) | 1 to 50 |
| Treatment received |  |
| Seeking advice from a medical doctor (consultation) | 119 (68.39) |
| Undergoing fertility diagnostic testing (diagnosis) | 51 (29.31) |
| Ovulation induction | 11 (6.32) |
| Insemination | - |
| Surgery | - |
| Assisted Reproduction Technology (ART) | - |
| Drugs | 147 (84.48) |
| Counselling | 41 (23.56) |
| None of these | - |
| Others (folk, traditional and herbal remedies) | 3 |
| **Specialist doctor** | |
| Number of special doctors consulted | 1009 (94.12) |
| Mean (SD) specialist doctors consulted | 2.22 (1.41) |
| Number of times each specialist doctor consulted | |
| Median (IQR) | 13 (5 to 33) |
| Range | 1 to 212 |
| Treatment received |  |
| Seeking advice from a medical doctor (consultation) | 788 (78.10) |
| Undergoing fertility diagnostic testing (diagnosis) | 924 (91.58) |
| Ovulation induction | 456 (45.19) |
| Insemination | 45 (4.46) |
| Surgery | 34 (3.37) |
| Assisted Reproduction Technology (ART) | 14 (1.39) |
| Drugs | 950 (94.15) |
| Counselling | 64 (6.34) |
| None of these | - |
| Others | 4 (0.40) |
| **Parent (s)** | |
| Number of parents consulted | 457 (42.63) |
| Mean (SD) parent(s) consulted | 1.01 (0.11) |
| Number of times each parent(s) consulted | |
| Median (IQR) | 1 (1 to 2) |
| Range | 1 to 60 |
| Treatment received |  |
| Seeking advice from a medical doctor (consultation) | - |
| Undergoing fertility diagnostic testing (diagnosis) | - |
| Ovulation induction | - |
| Insemination | - |
| Surgery | - |
| Assisted Reproduction Technology (ART) | - |
| Drugs | 1 |
| Counselling | 455 (99.56) |
| None of these | - |
| Others (folk, traditional and herbal remedies) | 1 |
| **Family members (non-parent)** | |
| Number of family members consulted | 628 (58.58) |
| Mean (SD) family members (non-parent) consulted | 1.16 (0.46) |
| Number of times each family member (non-parent) consulted | |
| Median (IQR) | 1 (1 to 2) |
| Range | 1 to 50 |
| Treatment received |  |
| Seeking advice from a medical doctor (consultation) | - |
| Undergoing fertility diagnostic testing (diagnosis) | - |
| Ovulation induction | - |
| Insemination | - |
| Surgery | - |
| Assisted Reproduction Technology (ART) | - |
| Drugs | 1 |
| Counselling | 625 (99.52) |
| None of these | - |
| Others (folk, traditional and herbal remedies) | - |
| **Religious leader (Prayer or medication)** | |
| Number of religious leaders (Prayer or medication) consulted | 371 (34.61) |
| Mean (SD) religious leader (Prayer or medication) consulted | 1.74 (1.35) |
| Number of times each religious leader (Prayer or medication) consulted | |
| Median (IQR) | 2 (1 to 6) |
| Range | 1 to 90 |
| Treatment received |  |
| Drugs | 36 (9.70) |
| Counselling | 258 (69.54) |
| Others like advice on sources of treatment | 5 (1.35) |
| Others (folk, traditional and herbal remedies) | 231 (62.26) |
| **Accessed a help line** | |
| Number of helplines consulted | 13 (1.21) |
| Mean (SD) helpline consulted | 1.08 (0.28) |
| Number of times each helpline consulted | |
| Median (IQR) | 1 (1 to 1) |
| Range | 1 to 2 |
| Treatment received |  |
| Seeking advice from a medical doctor (consultation) | 3 (23.08) |
| Undergoing fertility diagnostic testing (diagnosis) | - |
| Ovulation induction | - |
| Insemination | - |
| Surgery | - |
| Assisted Reproduction Technology (ART) | - |
| Counselling | 10 (76.92) |
| None of these | - |
| Others | - |
| **Non-Governmental Organization** | |
| Number of non-governmental organizations consulted | 42 (3.92) |
| Mean (SD) non-governmental organizations consulted | 1.0 (0) |
| Number of times each non-governmental organization consulted | |
| Median (IQR) | 1 (1 to 1) |
| Range | 1 to 36 |
| Treatment received | |
| Seeking advice from a medical doctor (consultation) | 6 (14.29) |
| Undergoing fertility diagnostic testing (diagnosis) | 13 (30.95) |
| Ovulation induction | - |
| Insemination | - |
| Surgery | - |
| Assisted Reproduction Technology (ART) | - |
| Drugs | 11 (26.19) |
| Counselling | 31 (73.81) |
| None of these | 1 |
| **Government social services** | |
| Number of government social services consulted | 40 (3.73) |
| Mean (SD) government social services consulted | 1.18 (0.38) |
| Number of times each government social services consulted | |
| Median (IQR) | 5 (2 to 10) |
| Range | 1 to 85 |
| Treatment received | |
| Seeking advice from a medical doctor (consultation) | 23 (57.50) |
| Undergoing fertility diagnostic testing (diagnosis) | 22 (55.0) |
| Ovulation induction | 3 (7.50) |
| Insemination | - |
| Surgery | 1 |
| Assisted Reproduction Technology (ART) | - |
| Drugs | 35 (87.50) |
| Counselling | 6 (15.0) |
| **Friend (s)** | |
| Number of friends consulted | 88 (8.21) |
| Mean(SD) friends consulted | 1.02 (0.21) |
| Mean(SD) number of times each friend consulted |  |
| Median (IQR) | 1 (1 to 1) |
| Range | 1 to 50 |
| Treatment received | |
| Seeking advice from a medical doctor (consultation) | 1 |
| Counselling | 87 (98.86) |
| **Government Health system** | |
| Number of government health systems consulted | 8 (0.01) |
| Mean (SD) government health systems consulted | 1 (0) |
| Number of times each government health system consulted |  |
| Median (IQR) | 1 (1 to 1) |
| Range | 1 to 3 |
| Treatment received | |
| Seeking advice from a medical doctor (consultation) | 1 |
| Undergoing fertility diagnostic testing (diagnosis) | 1 |
| Ovulation induction | - |
| Insemination | - |
| Surgery | - |
| Assisted Reproduction Technology (ART) | - |
| Drugs | 2 (25.0) |
| Counselling | 6 (75.0) |
| None of these | - |
| Others | - |
| **Study staff** | |
| Number of study staff consulted | 172 (16.04) |
| Mean (SD) study staff consulted | 1.09 (0.35) |
| Number of times each study staff consulted | |
| Median (IQR) | 2 (1 to 3) |
| Range | 1 to 18 |
| Treatment received | |
| Seeking advice from a medical doctor (consultation) | 8 (4.65) |
| Undergoing fertility diagnostic testing (diagnosis) | 3 (1.74) |
| Ovulation induction | - |
| Insemination | - |
| Surgery | - |
| Drugs | 3 (1.74) |
| Counselling | 161 (93.60) |
| **Other (folk, traditional and herbal remedies)** | |
| Number of other remedies applied | 224 (20.90) |
| Mean (SD) other remedies applied | 1.20 (0.59) |
| Number of times each friend consulted |  |
| Median (IQR) | 2 (1 to 4) |
| Range | 1 to 40 |

**Supplementary Figure 1: Scree plot of eigen values after factor loading**

**
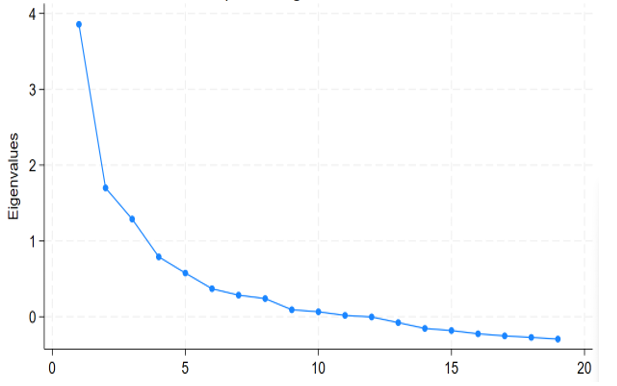
**

**Supplementary Table 2:** Multivariable model representing the determinants of care-seeking for delayed conception from all sources of care versus formal and mixed sources of care among study participants.

| **Variables** | **Sought Care** | |
| --- | --- | --- |
|  | **Adjusted OR (95% CI), (p-value)**  (Source: All types, including Formal, Informal and Mixed) | **Adjusted OR (95% CI), (p-value)**  Source: Only from Formal, and Mixed) |
| **DEMOGRAPHIC** | | |
| Women age |  |  |
| >= 18 to 21 years | Reference | Reference |
| >=22 to 27 years | 0.6 (0.5, 0.8) (p=0.000*) | 0.8 (0.6, 1.0) (p=0.05) |
| 28 years and above | 0.2 (0.1, 0.5) (p=0.001*) | 0.4 (.2 1.0) (p=0.07) |
| **FERTILITY INTENTIONS** | | |
| Perception of the woman that conception is taking longer  Yes  No | 1.6(1.4,1.7) (p=<0.001***)  Reference | 4.6 (3.2,6.6) (p=<0.001***)  Reference |
| Women felt isolated  Yes  No | 1.7(1.2,2.3) (p=<0.001***)  Reference | 2.4 (1.8,3.1) (p=<0.001***)  Reference |
| Women emotionally abused by partner/husband  Yes  No | 1.6** (1.2,2.4) (p=<0.005**)  Reference | 1.8 (1.4,2.4) (p=<0.001*)  Reference |
| Women emotionally abused by family members  Yes  No | 1.5 (1.2,1.8) (p=0.05*)  Reference | 1.8* (1.3,2.4) (p=<0.001**)  Reference |
| **MEDICAL HISTORY** | | |
| Regular period every month  Yes  No | 0.7*(0.4,0.9) (p=0.02*)  Reference | 0.6* (0.4,0.9) (p=0.01*)  Reference |
| Bleeding Heavy during periods  Yes  No | 1.5 (1.0,2.2) (p=0.03*)  Reference | - |
